# Xylanase production from *Penicillium citrinum* isolate HZN13 using response surface methodology and characterization of immobilized xylanase on glutaraldehyde-activated calcium-alginate beads

**DOI:** 10.1007/s13205-016-0484-9

**Published:** 2016-08-11

**Authors:** Zabin K. Bagewadi, Sikandar I. Mulla, Yogesh Shouche, Harichandra Z. Ninnekar

**Affiliations:** 1 Department of Biochemistry, Karnatak University, Dharwad, 580 003 Karnataka India; 2 National Centre for Cell Science, Pune University Campus, Ganeshkhind, Pune, 411 007 Maharashtra India

**Keywords:** *Penicillium citrinum*, Xylanase, Sweet sorghum bagasse, Response surface methodology, Immobilization, Enzymatic hydrolysis

## Abstract

The present study reports the production of high-level cellulase-free xylanase from *Penicillium citrinum* isolate HZN13. The variability in xylanase titers was assessed under both solid-state (SSF) and submerged (SmF) fermentation. SSF was initially optimized with different agro-waste residues, among them sweet sorghum bagasse was found to be the best substrate that favored maximum xylanase production (9643 U/g). Plackett–Burman and response surface methodology employing central composite design were used to optimize the process parameters for the production of xylanase under SSF. A second-order quadratic model and response surface method revealed the optimum conditions for xylanase production (sweet sorghum bagasse 25 g/50 ml; ammonium sulphate 0.36 %; yeast extract 0.6 %; pH 4; temperature 40 °C) yielding 30,144 U/g. Analysis of variance (ANOVA) showed a high correlation coefficient (*R* ^2^ = 97.63 %). Glutaraldehyde-activated calcium-alginate-immobilized purified xylanase showed recycling stability (87 %) up to seven cycles. Immobilized purified xylanase showed enhanced thermo-stability in comparison to immobilized crude xylanase. Immobilization kinetics of crude and purified xylanase revealed an increase in *K*_m_ (12.5 and 11.11 mg/ml) and *V*_max_ (12,500 and 10,000 U/mg), respectively. Immobilized (crude) enzymatic hydrolysis of sweet sorghum bagasse released 8.1 g/g (48 h) of reducing sugars. Xylose and other oligosaccharides produced during hydrolysis were detected by High-Performance Liquid Chromatography. The biomass was characterized by Scanning Electron Microscopy, Energy Dispersive X-ray and Fourier Transformation Infrared Spectroscopy. However, this is one of the few reports on high-level cellulase-free xylanase from *P. citrinum* isolate using sweet sorghum bagasse.

**Electronic supplementary material:**

The online version of this article (doi:10.1007/s13205-016-0484-9) contains supplementary material, which is available to authorized users.

## Introduction

Xylan is the major structural polysaccharide constituent of hard and soft wood and is the second most abundant renewable resource. This complex heteropolysaccharide is composed of β-(1,4)-linked d-xylopyranosyl residues with substitutions of l-arabinofuranose, d-glucuronic acid, and 4-*O*-methyl-d-glucuronic acid. Xylan degradation requires different xylanolytic enzymes, like xylanase (EC 3.2.1.8), β-xylosidase (EC 3.2.1.37), α-l-arabinofuranosidase (EC 3.2.1.55), α-d-glucuronidase (EC 3.2.1.139), and acetyl xylan esterase (EC 3.1.1.72) (Beg et al. 2001). Crude enzyme preparations are cost effective as they bypass the high cost involved in downstream processing. Such preparations have been employed as cocktails for enzymatic hydrolysis of biomass which requires a pool of hemicellulolytic enzymes. A mixture of crude extracts from *Trichoderma viride* and commercial cellulolytic enzymes have been used to hydrolyze cellulose Avicel (Vintila et al. 2010). Crude xylanase have been used for the hydrolysis of xylan and hemicellulosic materials to several xylooligosaccharides. However, xylanase with specific characteristics like pH and thermo-stability, high specific activity, resistance to metal ions and chemicals are required to meet the desired needs of industries (Ramírez-Cavazos et al. 2014), which could be achieved by enzyme purification. Purified xylanases have been employed in feedstuff (Knob and Carmona 2010) and cellulase-free xylanase has gained importance in paper and pulp industry (Collins et al. 2005). Highly purified xylanase finds specific applications in synthetic chemistry, food and cosmetic industries, medical diagnostics (Rodriguez Couto and Toca Herrera 2006) and acts as inhibitory agent towards human HIV-1 reverse transcriptase (Xiao et al. 2003). Enzyme purification is essential for the determination of biochemical, molecular, accurate kinetic, structural and functional properties. Because, it helps to understand the molecular interactions, secondary structures of proteins and also reveals the occurrence of multiple isoforms of enzymes. Based on the amino acid sequence of purified enzymes, they have been classified into glycosyl hydrolase families (Henrissat and Davies 1997). For commercial production of enzymes, the focus is on utilization of agro-residual wastes along with development of efficient bioprocess strategies to obtain high enzyme titers. Hence, lot of emphasis has been given for screening of such agricultural residues like rice straw, wheat straw, and sugarcane bagasse. Moreover, sweet sorghum bagasse could be a potential substrate for production of higher enzyme titers. Xylanase has been reported from microbial sources like *Aspergillus* sp. and *Trichoderma* sp., as well as bacterial isolates (Sapag et al. 2002). However, less work on xylanase from *Penicillium citrinum* isolate has been reported and, moreover, cellulase-free xylanases are of considerable research interests due to their industrial significance (Walia et al. 2014). An attractive fermentation process is SSF for xylanase production as it involves the growth of fungi on moist substrates in the absence of free flowing water thereby mimicking the natural environment. Due to low water content in SSF, the microbe is in contact with gaseous oxygen and substrate, unlike in the case of SmF. Therefore, SSF offers several advantages over SmF, such as compactness, higher product yields, less investment and low energy demand. Hence, SSF has been widely employed in enzyme production, solid waste management, biomass energy conversion and in production of microbial secondary metabolites (Holker et al. 2004; Narang et al. 2001). To develop a successful fermentation process, one of the approaches is to optimize the process parameters to improve enzyme yields (El-Hadi et al. 2014). There are two ways by which optimization of fermentation process can be addressed: classical and statistical. The classical approach is based on the “one-factor-at-a-time” in which one independent variable is studied while fixing all the other factors at a constant level (Khucharoenphaisan et al. 2008), but this method seems to be time consuming, gives unreliable results and inaccurate conclusion. Hence, an alternate strategy is statistical experimental designs including Plackett–Burman design (PBD) and response surface methodologies (RSM) which can collectively eliminate these limitations of a single-factor optimization process and has been extensively used for optimization of fermentation factors for enzyme production using SSF (Trivedi et al. 2012). RSM involves a minimum number of experiments for a large number of variables and simultaneously solves multivariate equations, by which improvement in enzyme production has been demonstrated successfully (Khucharoenphaisan et al. 2008). RSM has been employed for modeling and optimization of process parameters for enzyme production, wastewater treatment as well as production of extracellular polysaccharides (Zambare and Christopher 2011) and many in biochemical as well as biotechnological processes (Bas and Boyaci 2007). Although RSM has been used to optimize the production of microbial xylanases, less work has been reported on xylanase production by SSF using *P. citrinum* isolate. Enzyme immobilization offers advantages like reusability and continuous processing. Enzyme immobilization methods vary for different enzymes and applications. Covalent immobilization of xylanase on glutaraldehyde-activated calcium-alginate beads has proved to be easy and economical (Pal and Khanum 2011). Enzymatic hydrolysis using immobilized enzymes has been demonstrated to produce xylooligosaccharides (Aragon et al. 2013b).

Here, we report the characterization of a fungal isolate for the production of high-level cellulase-free xylanase from a variety of agro-waste residues. The isolated *P. citrinum* isolate HZN13 was used for xylanase production through a sequentially designed PBD and RSM statistical process optimization using sweet sorghum bagasse. Crude and purified xylanase was immobilized on glutaraldehyde-activated calcium-alginate beads and characterized. The crude immobilized xylanase was employed for enzymatic hydrolysis of bagasse. Reports on high-level xylanase from *P. citrinum* isolate induced by sweet sorghum bagasse are scare.

## Materials and methods

### Chemicals and substrates

All the chemicals used in the present study were purchased from HiMedia Laboratories Ltd., Sigma-Aldrich Pvt Ltd. (USA) and Merck and Co. Inc. (USA). Sweet sorghum stalks (SS), wheat bran (WB), sugarcane bagasse (SB), rice bran (RB), corn cobs (CC), and saw dust (SD) were collected from fields and local market.

### Isolation and screening of xylanolytic fungi

A cellulase-free xylanase-producing fungal isolate HZN13 was isolated from composting forest soil. Soil samples were suspended in sterile distilled water and serially diluted samples were spread on Mandel’s and Weber media (Szijártó et al. 2004) supplemented with 1 % Birchwood xylan. Plates were incubated at 30 °C for 5–10 days. Morphologically diverse colonies were isolated and purified by repeated streaking. Pure cultures were preserved on potato dextrose agar (PDA) slants at 4 °C. Xylanase producers were screened on selective xylan agar media containing (g L^−1^): NaNO_3_, 2.0; KH_2_PO_4_, 1.0; MgSO_4_⋅7H_2_O, 0.5; KCl, 0.5; xylan, 10.0; peptone, 0.2 and agar, 17.0 in distilled water; pH 5.0. Inoculated plates were incubated at 35 °C for 3–4 days. The plates were flooded with 1 % (w/v) Congo red for 15 min followed by 10 min of destaining with 1 M NaCl and subsequently the diameter of zone of clearance was measured. Based on greater zone of clearance, HZN13-positive isolate was selected and characterized based on morphology, reproductive structures and microscopy (Robl et al. 2013).

### Molecular characterization by 18S rDNA gene sequence analysis

The isolated pure fungal culture was molecularly characterized by 18S rDNA sequencing followed by phylogenetic analysis. Fungal DNA was isolated from fresh mycelia grown on PDA by methods described previously (Singh et al. 2010). PCR amplification of ribosomal internal transcribed spacer (ITS) region of genomic DNA was carried out using primers ITS1 (5′-GCGGATCCGTAGGTGAACCTGCGG) and ITS4 (5′-GCGGATCCTCCGCTTATT GATATGC) (Singh et al. 2011). PCR products were gel purified (Sigma, Genosys, USA) and sequenced by Big Dye Terminator cycle sequencing kit (V3.1, Applied Biosystems, USA) according to the manufacturer’s procedure and analyzed in a DNA Analyzer (3730 DNA Analyzer, Applied Biosystems, USA). Sequence data were edited using Chromas Pro version 1.34. Sequence was analyzed using BLASTn program (http://www.ncbi.nlm.nih.gov/) and aligned using Clustal*W* (http://www.ebi.ac.uk/Tools/msa/clustalo/) (Singh et al. 2012). The phylogenetic tree was constructing using distance setting by MEGA 6 software with 100 bootstrap replicates (Tamura et al. 2013), by the neighbor-joining (NJ) method (Kumar et al. 2008).

### Production of xylanase in SmF and SSF

Xylanase was produced under both SmF and SSF. The agricultural residues (SS, WB, SB, RB, CC and SD) used in the fermentation processes were dried, powdered and subjected to alkali (4 % NaOH) pretreatment. These pretreated substrates were used for xylanase production by *P. citrinum* isolate HZN13. SmF was carried out in 250-ml Erlenmeyer flasks using pretreated substrates in modified Mandels–Weber medium containing (g/L) urea 0.3; ammonium sulfate 1.4; KH_2_PO_4_ 0.3; CaCl_2_ 0.3; MgSO_4_⋅7H_2_O 0.3; yeast extract 1.0 and (mg/L) FeSO_4_⋅7H_2_O 5.0; MnSO_4_⋅7H_2_O 1.6; ZnSO_4_⋅7H_2_O 1.4; CoCl_2_ 2; Tween-80 0.1 % (pH 5). Inoculums were added to the culture medium and incubated at 30 °C in a rotary shaker (200 rpm) for 4 days. Fungal biomass was separated by vacuum filtration (Millipore India Ltd.). The filtrate was centrifuged at 8000×*g* for 20 min at 4 °C and the supernatant was assayed for xylanase activity. SSF was carried out with 10 g of substrate moistened with modified Mandels–Weber medium to attain a final moisture level of 70 % and incubated under static condition for 6 days followed by enzyme extraction with citrate buffer (50 mM, pH 4 and 1:2 solid to liquid ratio) under shaking (150 rpm) at 30 °C for 30 min. Biomass was separated as mentioned above and samples were used for xylanase assay and protein analysis.

### Enzyme assays and protein determination

Xylanase activity was estimated by the modified method of Bailey et al. (1992) using 1 % (w/v) Birchwood xylan in citrate buffer (pH 4) at 55 °C for 30 min. Cellulase activity was calculated using 1 % (w/v) carboxymethyl cellulose (CMC) by methods described previously (Bagewadi et al. 2016). The reducing sugars in the reactions were determined by Miller (1959) method. One unit (U) of enzyme activity was defined as the amount of enzyme capable to release 1 μmol of the reducing sugars (glucose or xylose equivalent) from the substrate in 1 min under standard assay conditions. Protein was estimated by bicinchoninic acid (BCA) protein assay kit (Mulla et al. 2016). Assays were carried out in triplicate, and the data are represented as mean ± standard deviation.

### SDS-PAGE and zymogram analysis

The xylanase produced from sweet sorghum bagasse under SmF and SSF was compared by sodium dodecylsulphate–polyacrylamide gel electrophoresis (SDS-PAGE) by the method of Laemmli (1970). Protein bands were detected by staining with silver nitrate. The zymogram analysis of xylanase was performed by the method of Driss et al. (2012) with slight modifications. The protein separation was carried out at 4 °C by native PAGE electrophoresis with 10 % polyacrylamide gel using TBE buffer. After the completion of electrophoresis, the gel was washed twice with 25 % (v/v) isopropanol in citrate buffer (50 mM, pH 4) and incubated in the same buffer containing 1 % Birchwood xylan. The gel was then stained with 0.5 % (w/v) Congo red solution containing 5 % (v/v) ethanol to detect the xylanase activity. The gel was destained with 1 M NaCl solution. Clear bands against a red background signified xylanase activity.

### Screening of significant factors by PBD 

Screening of significant factors influencing xylanase production from a large set of variables was carried out by PBD with minimum experimental runs (Rajendran et al. 2007). The independent variables selected for the design were urea (*X*_1_), ammonium sulfate (*X*_2_), KH_2_PO_4_ (*X*_3_), CaCl_2_ (*X*_4_), MgSO_4_⋅7H_2_O (*X*_5_), yeast extract (*X*_6_) and sweet sorghum bagasse (*X*_7_). Other media components were kept constant. Seven variables were screened with 12 experimental trails in duplicates at two levels, high (+1) and low (−1). The actual and coded form of high and low values of variables and the complete experimental design matrix with response is shown in Table 1. The response is the average of xylanase production (U/g). PBD is based on the first-order polynomial model (Eq. 1):

1 $$Y = \beta_{o} + \varSigma \beta_{i} X_{i}$$

where *Y* is the response (xylanase production U/g), *β*_*o*_ is the model intercept, *β*_*i*_ is the linear coefficient and *X*_*i*_ is the level of the independent variable (*i* = 1, 2, 3, 4, 5, 6 and 7).

**Table 1:** PBD matrix for the screening of independent variables with actual and coded values affecting xylanase production

Run no.	*X*_1_ urea (%)	*X*_2_ ammonium sulphate (%)	*X*_3_ KH_2_PO_4_ (%)	*X*_4_ CaCl_2_ (%)	*X*_5_ MgSO_4_⋅H_2_O (%)	*X*_6_ yeast extract (%)	*X*_7_ sweet sorghum bagasse (g/50 ml)	Xylanase production (U/g)	
								Experimental	Predicted
1	0.6 (+1)	0.14 (−1)	0.06 (+1)	0.03 (−1)	0.03 (−1)	0.1 (−1)	10 (+1)	13,128	13,811.3
2	0.6 (+1)	0.28 (+1)	0.03 (−1)	0.06 (+1)	0.03 (−1)	0.1 (−1)	5 (−1)	9896	9945
3	0.3 (−1)	0.28 (+1)	0.06 (+1)	0.03 (−1)	0.06 (+1)	0.1 (−1)	5 (−1)	9100	9456.3
4	0.6 (+1)	0.14 (−1)	0.06 (+1)	0.06 (+1)	0.03 (−1)	0.2 (+1)	5 (−1)	10,740	10,761
5	0.6 (+1)	0.28 (+1)	0.03 (−1)	0.06 (+1)	0.06 (+1)	0.1 (−1)	10 (+1)	17,654	17,605
6	0.6 (+1)	0.28 (+1)	0.06 (+1)	0.03 (−1)	0.06 (+1)	0.2 (+1)	5 (−1)	12,890	12,533.7
7	0.3 (−1)	0.28 (+1)	0.06 (+1)	0.06 (+1)	0.03 (−1)	0.2 (+1)	10 (+1)	20,250	19,832
8	0.3 (−1)	0.14 (−1)	0.06 (+1)	0.06 (+1)	0.06 (+1)	0.1 (−1)	10 (+1)	15,630	15,343.7
9	0.3 (−1)	0.14 (−1)	0.03 (−1)	0.06 (+1)	0.06 (+1)	0.2 (+1)	5 (−1)	11,321	12,004.3
10	0.6 (+1)	0.14 (−1)	0.03 (−1)	0.03 (−1)	0.06 (+1)	0.2 (+1)	10 (+1)	18,480	18,132
11	0.3 (−1)	0.28 (+1)	0.03 (−1)	0.03 (−1)	0.03 (−1)	0.2 (+1)	10 (+1)	19,125	19,543
12	0.3 (−1)	0.14 (−1)	0.03 (−1)	0.03 (−1)	0.03 (−1)	0.1 (−1)	5 (−1)	8148	7394.7

### Process optimization by RSM

A central composite design (CCD) under RSM was employed for optimization to obtain maximum xylanase production after selecting significant factors from PBD using Minitab 17 statistical software. The five independent variables selected were sweet sorghum bagasse (*A*), yeast extract (*B*), ammonium sulphate (*C*), pH (*D*) and temperature (*E*) at five coded levels (−α, −1, 0, +1 +α) as shown in Table 2. Other media components were maintained constant. The relation between the coded forms of the input variable and the actual value of sweet sorghum bagasse, yeast extract, ammonium sulphate, pH and temperature are described in Eq. (2):

2 $$frac{{X_{i} = (Z_{i} {-}Z_{0} )}}\div{\Delta Z}$$

where *X*_*i*_ is a coded value and *Z*_*i*_ the actual value of the variable, *Z*_0_ is the actual value of the same variable at the center point, Δ*Z* is the step change of the variable. The full experimental plan of selected variables with 52 experimental trails in replicates, under predefined conditions is shown in Table 3. The response (xylanase production U/g) represents the combined effects of five independent variables studied in a defined range. The xylanase production U/g (*Y*) as response was fitted using a second-order polynomial (Eq. 3):

3 $$\begin{aligned} Y & = \beta_{o} + \beta_{1} A + \beta_{2} B + \beta_{3} C + \beta_{4} D + \beta_{5} E + \beta_{11} A^{2} + \beta_{22} B^{2} + \beta_{33} C^{2} \\ & \quad + \beta_{44} D^{2} + \beta_{55} E^{2} + \beta_{12} AB + \beta_{13} AC + \beta_{14} AD + \beta_{15} AE + \beta_{23} BC \\ & \quad + \beta_{24} BD + \beta_{25} BE + \beta_{34} CD + \beta_{35} CE \\ \end{aligned}$$

where *Y* is the measured response (xylanase production U/g), *A*, *B*, *C*, *D* and *E* are independent variables, *β*_1_, *β*_2_, *β*_3_, *β*_4_, *β*_5_ are linear coefficients, *β*_11_, *β*_22_, *β*_33_, *β*_44_, *β*_55_ are quadratic coefficients and *β*_12_, *β*_13_, *β*_14_, *β*_15_,*β*_23_, *β*_24_, *β*_25_, *β*_34_, *β*_35_ are cross product coefficients of the model.

**Table 2:** Actual and coded level of independent variables tested with RSM-CCD design for xylanase production

Independent process variable	Symbol code	Actual values of coded variables				
		+α	+1	0	−1	−α
Sweet sorghum bagasse (g/50 ml)	*A*	31.85	25	20	15	8.15
Yeast extract (%)	*B*	1.075	0.8	0.6	0.4	0.125
Ammonium sulphate (%)	*C*	0.3675	0.34	0.32	0.30	0.2725
pH	*D*	5.185	4.5	4.0	3.5	2.815
Temperature (°C)	*E*	46.5	40	35	30	23.5

**Table 3:** Experimental design matrix of RSM-CCD of significant variables affecting xylanase production

Run no.	*A* sweet sorghum bagasse (g/50 ml)	*B* yeast extract (%)	*C* ammonium sulphate (%)	*D* pH	*E* temperature (°C)	Xylanase production (U/g)	
						Experimental	Predicted
1	−1	−1	−1	−1	−1	8742	7756.3
2	+1	−1	−1	−1	−1	18,964	17,080.2
3	−1	+1	−1	−1	−1	6923	5419.9
4	+1	+1	−1	−1	−1	12,010	13,189.9
5	−1	−1	+1	−1	−1	8614	9386.2
6	+1	−1	+1	−1	−1	22,246	23,500.2
7	−1	+1	+1	−1	−1	7746	8622.2
8	+1	+1	+1	−1	−1	22,135	21,182.3
9	−1	−1	−1	+1	−1	7612	9131
10	+1	−1	−1	+1	−1	8221	9884.3
11	−1	+1	−1	+1	−1	6915	8534.2
12	+1	+1	−1	+1	−1	8000	7733.6
13	−1	−1	+1	+1	−1	9433	8998.6
14	+1	−1	+1	+1	−1	15,144	14,542
15	−1	+1	+1	+1	−1	9600	9974.2
16	+1	+1	+1	+1	−1	13,402	13,963.7
17	−1	−1	−1	−1	+1	5426	6577.9
18	+1	−1	−1	−1	+1	17,237	17,894.4
19	−1	+1	−1	−1	+1	8961	7720.3
20	+1	+1	−1	−1	+1	16,112	17,483
21	−1	−1	+1	−1	+1	10,824	9445.2
22	+1	−1	+1	−1	+1	26,432	25,551.8
23	−1	+1	+1	−1	+1	10,210	12,160
24	+1	+1	+1	−1	+1	29,100	26,712.8
25	−1	−1	−1	+1	+1	7100	6419.2
26	+1	−1	−1	+1	+1	9314	9165.1
27	−1	+1	−1	+1	+1	6954	9301.3
28	+1	+1	−1	+1	+1	12,122	10,493.4
29	−1	−1	+1	+1	+1	5300	7524.2
30	+1	−1	+1	+1	+1	14,216	15,060.2
31	−1	+1	+1	+1	+1	13,628	11,978.6
32	+1	+1	+1	+1	+1	14,348	17,960.8
33	−2.37841	0	0	0	0	7005	5815.3
34	+2.37841	0	0	0	0	24,128	24,017.4
35	0	−2.37841	0	0	0	8510	8106.3
36	0	+2.37841	0	0	0	9674	8777.3
37	0	0	−2.37841	0	0	8865	8428
38	0	0	+2.37841	0	0	20,110	19,246.7
39	0	0	0	−2.37841	0	9012	10,748.9
40	0	0	0	+2.37841	0	5013	1975.8
41	0	0	0	0	−2.37841	7505	7059.3
42	0	0	0	0	+2.37841	11,266	10,411.3
43	0	0	0	0	0	28,240	28,132.4
44	0	0	0	0	0	28,380	28,132.4
45	0	0	0	0	0	28,150	28,132.4
46	0	0	0	0	0	28,756	28,132.4
47	0	0	0	0	0	28,642	28,132.4
48	0	0	0	0	0	28,120	28,132.4
49	0	0	0	0	0	28,010	28,132.4
50	0	0	0	0	0	28,000	28,132.4
51	0	0	0	0	0	27,980	28,132.4
52	0	0	0	0	0	27,900	28,132.4

The Minitab 17 statistical software was used for analyzing the experimental data. Statistical analysis, three-dimensional (3D) surface plots and contour plots of the model were also done using same software. ANOVA was used to establish the statistical significance of the model terms. Overall model significance was evaluated using Fisher’s ‘*F*’ test and its corresponding probability ‘*p*’. The quality of the model equation was assessed statistically by coefficient of determination *R* ^2^ and adjusted *R* ^2^. To validate the model and its prediction, experiments in triplicates were executed with the optimized levels of factors. Xylanase activity was measured as described previously.

### Immobilization of xylanase

The Ca-alginate beads were prepared by dropping 2 % (w/v) sodium alginate through a syringe into 0.2 M CaCl_2_ solution. The Ca-alginate beads were subjected for hardening by storing overnight in the same solution at 4 °C. Activation of Ca-alginate beads was carried out by immersing the beads in 8 % (w/v) glutaraldehyde solution in citrate buffer (50 mM, pH 4.0) under shaking (120 rpm) for 3 h. The beads were separated by filtration and washed repeatedly with distilled water to remove the unbound glutaraldehyde which was monitored at 245 nm. The activated beads were immersed separately in crude xylanase (15,000 U/ml) and purified xylanase (6900 U/ml) (data not shown) for immobilization under shaking (150 rpm) for 1 h. The immobilized beads were separated and washed repeatedly with distilled water until no xylanase activity was detected in the washings (Pal and Khanum 2011).

### Stability of immobilized xylanase

Recycling stability of glutaraldehyde-activated calcium-alginate immobilized crude and purified xylanase was assessed by determining the xylanase activity as mentioned above. The immobilized beads were separated after each reaction and washed with citrate buffer (50 mM, pH 4) repeatedly. Beads were reused for next reaction to hydrolyze Birchwood xylan up to seven reaction cycles. Xylanase activity in first cycle was considered as 100 %.

### Characterization of immobilized xylanase

pH optima of the immobilized crude and purified xylanase was evaluated using various buffer systems (pH 3–11) as described previously (Bagewadi et al. 2016). Immobilized xylanase stability at various pH (3–11) was evaluated by pre-incubating the beads in respective buffers for 2 h. The optimum temperature for immobilized crude and purified xylanase was determined between 20 and 75 °C. The thermo-stability of immobilized crude and purified xylanase was assessed by pre-incubating the beads for 2 h at respective temperatures (20–75 °C). The residual activity (%) was considered as 100 % at optimum pH and temperature. The kinetic parameters *K*_m_ and *V*_max_ of immobilized crude and purified xylanase were determined using Birchwood xylan by Lineweaver–Burk double reciprocal plot.

### Enzymatic hydrolysis by immobilized xylanase

Saccharification of alkali-pretreated sweet sorghum bagasse was carried out using glutaraldehyde-activated calcium-alginate immobilized crude xylanase produced by *P. citrinum* isolate HZN13. Hydrolysis reaction was carried out by the procedure described previously (Bagewadi et al. 2016). Briefly, the mixture containing substrate (bagasse) in citrate buffer (50 mM, pH 4) and immobilized crude xylanase was incubated at 50 °C under shaking (150 rpm). Aliquots were collected at regular time intervals and centrifuged to obtain clear supernatants for analysis of reducing sugars by Miller (1959) method. The immobilized beads were reused for five consecutive cycles of enzymatic hydrolysis. Controls used were reactions with denatured enzyme beads. Production of xylose during enzymatic hydrolysis was confirmed by HPLC.

### Analytical methods

The morphological changes of *P. citrinum* isolate HZN13 hydrolyzed sweet sorghum bagasse sample was investigated by SEM (VEGA\TESCAN, USA) according to the method described previously (Bagewadi et al. 2016). A careful observation was carried out with a consistent number of images from various sections at different magnification. The elemental analysis of the sample (*P. citrinum* isolate HZN13 hydrolyzed sweet sorghum bagasse) was carried out by the energy dispersive microanalysis system attached to SEM. Elemental composition was detected by X-ray analysis in EDX analyzer, where X-ray was emitted by the electron dense particles of sample following bombardment by electron beams. The treated sample was compared to the control sample (untreated sweet sorghum bagasse) from previously published report (Bagewadi et al. 2016).

FTIR (Perkin Elmer, FTIR1760) was employed to study the chemical group changes in *P. citrinum* isolate HZN13-hydrolyzed sweet sorghum bagasse sample by method described previously and the treated sample was compared to the control sample (untreated sweet sorghum bagasse) from previously published report (Bagewadi et al. 2016). Background scanning and correction were done prior to the acquisition of the spectrum.

The product (xylose) after enzymatic hydrolysis of sweet sorghum bagasse was analyzed by HPLC. HPLC (Dionex DX-600 series) used for the quantification of sugars consisted of an automated injector, degassing system, gradient pump, oven and UV detector. Chromatographic separation was achieved using a 150 × 4.6 mm S-3 column. Gradient elution using aqueous 0.05 % (v/v) phosphoric acid (pH 2–2.3) and water:acetonitrile (10:90) as mobile phase was carried out. 25 µL of sample was injected into the system with column temperature maintained at 30 °C and a flow rate of 1 ml/min was set. The detection wavelength was 210 nm. The xylose sugars were eluted out with gradients. Their retention time was compared with that of standard xylose.

## Results and discussion

### Isolation and molecular characterization by 18S rDNA gene sequence analysis

**Fig. 1:**
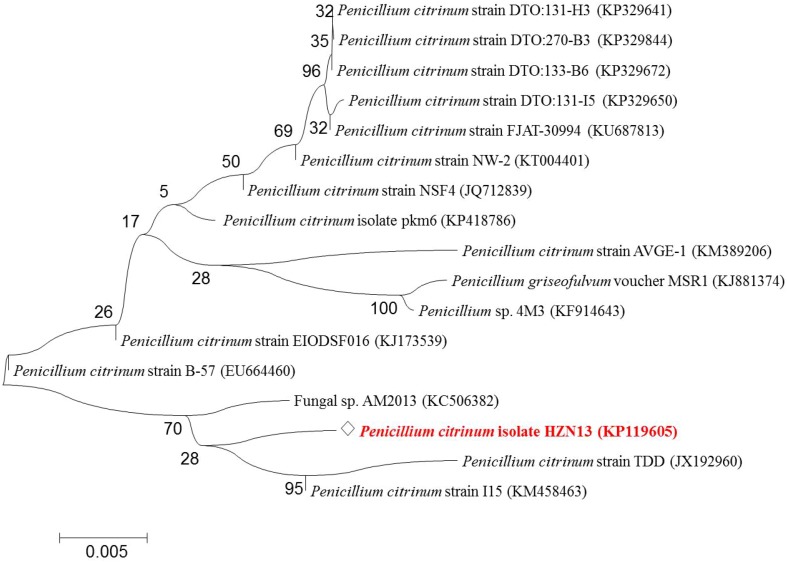
A consensus tree representing phylogenetic analysis of the 18S rDNA gene sequence analysis of isolated fungal culture [*diamond* *Penicillium citrinum* isolate HZN13 (Gene bank accession no. KP119605)]. Isolate sequence was used for BLASTn analysis in NCBI and the nearest neighbor sequences of other fungal cultures were chosen for Phylogenetic tree construction using MEGA 6 software with neighbor-joining method. *Numbers* at branches are bootstrap values of 100 replications

Among the isolated fungi isolates, HZN13 demonstrated to be a potential cellulase-free xylanse producer showing a zone of 2.8 cm after rapid screening with Congo red. Molecular identification (18S rDNA gene sequencing) was done on the basis of the sequence variation present in the ITS region. The ITS of HZN13 was amplified and sequenced. Sequence data were aligned and analyzed to identify the closest homologs with the submitted sequences in the national center for biotechnology information (NCBI) database. The phylogenetic tree was constructed by the NJ method with reference strains of *Penicillium* species from the NCBI GenBank. Phylogenetic analysis (Fig. 1) showed that isolate HZN13 belongs to the *P. citrinum* species and hence it was designated as *P. citrinum* isolate HZN13. The nucleotide sequence of the isolate has been deposited in NCBI GenBank database with the accession number KP119605. Xylanolytic potential of *P. citrinum* isolate HZN13 was assessed based on its growth and secretion of xylanase. The isolate grew profusely between 3^rd^ and 4^th^ day. Morphologically, the isolate showed grayish colonies. *Penicillium citrinum* isolate HZN13 was observed to possess globose conidia, septate hyphae and branched conidiophore after staining with lactophenol cotton blue. Several researchers have reported the isolation of xylanase-producing isolates like *Aspergillus tubingensis* FDHN1 from compost pit (Adhyaru et al. 2015), *Penicillium ramulosum* N1 from decaying wood (Lin et al. 2015), *Aspergillus fumigatus* isolate R1 (Deshmukh et al. 2016) and *P. citrinum* xym2 from garden soil (Saha and Ghosh 2014).

### Comparative production of cellulase-free xylanase in Smf and SSF

**Fig. 2:**
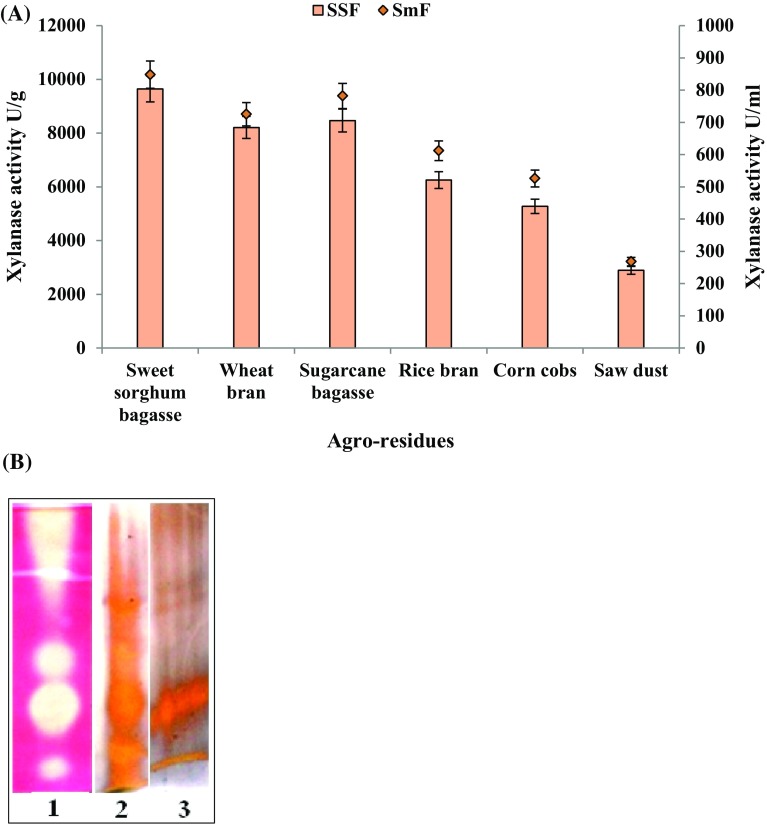
Production of cellulase-free xylanase in SmF and SSF using various agro-waste residues by *Penicillium citrinum* isolate HZN13 (**a**) and SDS-PAGE and zymogram analysis showing differential expression of xylanase from sweet sorghum bagasse in SmF and SSF [*lane 1* zymogram analysis for xylanase production in SSF; *lane 2* SDS-PAGE with silver nitrate staining of proteins produced in SSF; *lane 3* SDS-PAGE with silver nitrate staining of proteins produced in SmF] (**b**)

To assess the potential ability of *P. citrinum* isolate HZN13 for xylanase production, various agro-waste residues (SS, WB, SB, RB, CC and SD) as complex carbon source were evaluated by both Smf and SSF. All the substrates supported the fungal growth and induced the production of xylanase. During the fermentation processes, the highest yield of xylanase was 848 U/ml and 9643 U/g (Fig. 2a) in Smf and SSF, respectively, using sweet sorghum bagasse with a negligible amount of cellulase. Sugarcane bagasse and wheat bran also supported the production of high xylanase titers. Comparatively, xylanase yields were higher in SSF (2893–9643 U/g) than Smf (268–848 U/ml) from all the agro-waste residues. In addition, the proteins estimated were found to be higher in SSF (8.2–20.5 mg/gds) than Smf (0.22–0.71 mg/ml). The SDS-PAGE reveals the fact that higher enzyme titers are produced in SSF in comparison to SmF and shows the differential expression of proteins under varied fermentation modes (Fig. 2b). The protein bands were examined for their ability to hydrolyze the xylan incorporated into the gel. The zymogram analysis confirms the high-level xylanase production using sweet sorghum bagasse by *P. citrinum* isolate HZN13 in SSF (Fig. 2b). SSF also supported the expression of multiple forms of xylanases in comparison to SmF. Three distinct clear bands and smear in the regions of higher molecular weights indicate xylanase activity of crude extracts from SSF. Higher xylanase titers in SSF are attributed to the production of concentrated enzymes and similar natural habitat provided in SSF in comparison to SmF. In view of the higher yields, sweet sorghum bagasse was considered as a potential substrate and used throughout the optimization process and further studies. The high xylanase yield on sweet sorghum bagasse could be due to relatively high level of hemicelluloses and insoluble xylan content in this substrate. Structural variations in the architecture of the substrates induce differential levels of enzyme production. Xylanase production was found to be maximum around 4th day and 6th day in SmF and SSF, respectively. Thereafter, the gradually decline was observed, probably because the xylanases were degraded by proteolytic enzymes. These results suggest that the selection of an ideal natural substrate for xylanase production depends primarily on the availability of an inducing molecule and less quantity of anti-nutrients. Moreover, the high cost of pure xylan is not affordable for large-scale production of xylanase. Utilization of agro-waste residues is economical and beneficial as it takes care of solid waste disposal. Till date, studies on xylanase production using sweet sorghum bagasse as a substrate under SSF are scarce. Previously, use of sorghum straw has been reported for xylanase (615.5 U/g) production by *A. tubingensis* FDHN1 under SSF (Adhyaru et al. 2015). However, effective xylanase production using barley straw by *P. ramulosum* N1 has been reported (Lin et al. 2015). Similar to our results Pathak et al. (2014) also reported static solid state as the best fermentation type using wheat bran for enzyme production than SmF.

### Screening of significant factors affecting xylanase production using PBD

PB design was used to eliminate the insignificant factors from the design. Highest xylanase activity was 20,250 U/g (seventh trail). Large variation in xylanase production in different combinations indicates that the variations occurred due to the presence of different variables influencing the xylanase production at low and high levels (Table 1). Regression analysis and ANOVA with a *p* < 0.05 indicate the significance of the model terms and *p* values >0.1 indicate the insignificant model terms (Table 4). A significant positive effect of sweet sorghum bagasse, yeast extract and ammonium sulphate was demonstrated on xylanase production and sweet sorghum bagasse exhibited the maximum effect. The determination of coefficient *R* ^2^ and adjusted *R* ^2^ were 98.82 and 96.76 %, respectively, indicating a high correlation between the experimental and predicted values. The regression model describes the relationship between the independent variables and the response. The first-order model was fitted to the experimental results with the following equation:

4 $$Y = 1 3 8 6 4- { 65}X_{1} + 9 5 6X_{2} - 2 4 1X_{3} + 3 8 5X_{4} + 3 1 6X_{5} + 1 60 4X_{6} + 3 5 1 4X_{7}$$

where *Y* is predicted response and *X*_1_, *X*_2_, *X*_3_, *X*_4_, *X*_5_, *X*_6_ and *X*_7_ are the coded values of urea, ammonium sulphate, K_2_HPO_4_, CaCl_2_, MgSO_4_⋅7H_2_O, yeast extract and sweet sorghum bagasse, respectively.

**Table 4:** Regression coefficient and ANOVA for the quadratic model for xylanase production

Term	Effect	Coefficient	Degree of freedom	Adjusted sum of squares	Adjusted mean squares	*F* value	*p* value
Model			7	193,766,270	27,680,896	47.87	0.001*
Constant		13,864					<0.001*
*X*_1_—Urea	−131	−65	1	51,483	51,483	0.09	0.780
*X*_2_—Ammonium sulfate	1911	956	1	10,959,585	10,959,585	18.95	0.012*
*X*_3_—KH_2_PO_4_	−481	−241	1	694,083	694,083	1.20	0.335
*X*_4_—CaCl_2_	770	385	1	1,778,700	1,778,700	3.08	0.154
*X*_5_—MgSO_4_⋅7H_2_O	631	316	1	1,195,745	1,195,745	2.07	0.224
*X*_6_—Yeast extract	3208	1604	1	30,880,208	30,880,208	53.40	0.002*
*X*_7_—Sweet sorghum bagasse	7029	3514	1	148,206,465	148,206,465	256.29	<0.001*
Residual error			4	2,313,129	578,282		
Total			11	196,079,399			

*R* ^2^ = 98.82 %; Adjusted *R* ^2^ = 96.76 %; CV = 3.1

*F* Fisher’s function

Probability * (*p* < 0.05) corresponds to significance

### Optimization of xylanase production by RSM-CCD design

Significant variables from PBD were selected for optimization by RSM-CCD. The experimental responses for the 52 trials are presented in Table 3, which show considerable variation in xylanase production based on five independent variables in the medium. The minimum and maximum xylanase production achieved was 5300 and 28,756 U/g, respectively. The model was tested for adequacy by the ANOVA (Table 5). The computed *F* value (63.79) indicates that the model was significant at a high confidence level. The low probability *p* value (*p* < 0.05) of the model reflects the significance of the model. The high significance of the model was indicated by the correlation coefficient *R* ^2^ *=* 97.63 %. Moreover, *R* ^2^ is in reasonable agreement with the adjusted coefficient *R* ^2^ = 96.1 % indicating a high correlation between the experimentally observed and predicted values. However, 97.63 % of the variability could be explained by the model and 2.37 % of the total variations were not explained by the model. A smaller value of the coefficient of variation (CV) of 3.4 suggests a high level of precision in the obtained data. An insignificant value for the lack of fit indicated that the quadratic model was valid for the present study. The *p* < 0.05 for *A*, *C*, *D*, *E*, *A* ^2^, *B* ^2^, *C* ^2^, *D* ^2^, *E* ^2^, *AC*, *AD* and *BE* revealed the significance of these model terms (Table 5). The negative quadratic coefficient values for the variables suggest the existence of a peak point for xylanase production with respect to the variables and an inhibitory effect at other than the peak point. A positive linear coefficient value for *A*, *C*, *E* indicates increased xylanase production with increased concentrations of sweet sorghum bagasse and ammonium sulphate at higher temperatures. The combined effects of the process variables on the xylanase production could be expressed in the form of Eq. (5) where insignificant process variables have been excluded from the quadratic polynomial equation of the model.

**Table 5:** ANOVA for response surface quadratic model for xylanase production

Source	Coefficient	Degree of freedom	Sum of squares	Mean squares	*F* value	*p* value
Model		20	3,521,268,526	176,063,426	63.79	<0.001*
Linear		5	1,027,957,334	205,591,467	74.49	<0.001*
*A*—Sweet sorghum bagasse	3827	1	634,208,963	634,208,963	229.79	<0.001*
*B*—Yeast extract	141	1	861,752	861,752	0.31	0.580
*C*—Ammonium sulphate	2274	1	224,046,226	224,046,226	81.18	<0.001*
*D*—pH	−1844	1	147,332,061	147,332,061	53.38	<0.001*
*E*—Temperature	705	1	21,508,333	21,508,333	7.79	0.009*
Square		5	2,238,560,145	447,712,029	162.22	<0.001*
*A* ^2^—Sweet sorghum bagasse × sweet sorghum bagasse	−2336	1	319,451,969	319,451,969	115.75	<0.001*
*B* ^2^—Yeast extract × yeast extract	−3481	1	709,116,639	709,116,639	256.93	<0.001*
*C* ^2^—Ammonium sulphate × ammonium sulphate	−2527	1	373,743,394	373,743,394	135.42	<0.001*
*D* ^2^—pH × pH	−3848	1	866,803,762	866,803,762	314.06	<0.001*
*E* ^2^—Temperature × temperature	−3429	1	688,134,544	688,134,544	249.33	<0.001*
2-way interaction		10	254,751,047	25,475,105	9.23	<0.001*
*AB*—Sweet sorghum bagasse × yeast extract	−388	1	4,829,055	4,829,055	1.75	0.196
*AC*—Sweet sorghum bagasse × ammonium sulphate	1198	1	45,890,595	45,890,595	16.63	<0.001*
*AD*—Sweet sorghum bagasse × pH	−2143	1	146,911,226	146,911,226	53.23	<0.001*
*AE*—Sweet sorghum bagasse × temperature	498	1	7,941,109	7,941,109	2.88	0.100
*BC*—Yeast extract × ammonium sulphate	393	1	4,944,726	4,944,726	1.79	0.190
*BD*—Yeast extract × pH	435	1	6,052,590	6,052,590	2.19	0.149
*BE*—Yeast extract × temperature	870	1	24,205,143	24,205,143	8.77	0.006*
*CD*—Ammonium sulphate × pH	−441	1	6,211,931	6,211,931	2.25	0.144
*CE*—Ammonium sulphate × temperature	309	1	3,062,194	3,062,194	1.11	0.300
*DE*—pH × temperature	−383	1	4,702,478	4,702,478	1.70	0.201
Residual error		31	85,558,624	2,759,956	45.72	<0.001^a^
Lack of fit		22	84,799,892	3,854,541		
Pure error		9	758,732	84,304		
Total		51	3,606,827,149			

*R* ^2^ = 97.63 %; Adjusted *R* ^2^ = 96.1 %; Coefficient of variation (CV) = 3.4

*F* Fisher’s function

Probability *p* * (*p* < 0.05) corresponds to significance

^a^Insignificant

5 $$\begin{aligned} Y & = 28132 + 3827 \, A + 2274 \, C - 1844 \, D + 705 \, E - 2336 \, A^{2} - 3481 \, B^{2} - 2527 \, C^{2} \\ & \quad - 3848 \, D^{2} {-}3429 \, E^{2} + 1198 \, AC{-}2143 \, AD + 870 \, BE \\ \end{aligned}$$

where *Y* is the response (xylanase production U/g) and *A*, *B*, *C*, *D* and *E* are the coded values of the independent variables.

**Fig. 3:**
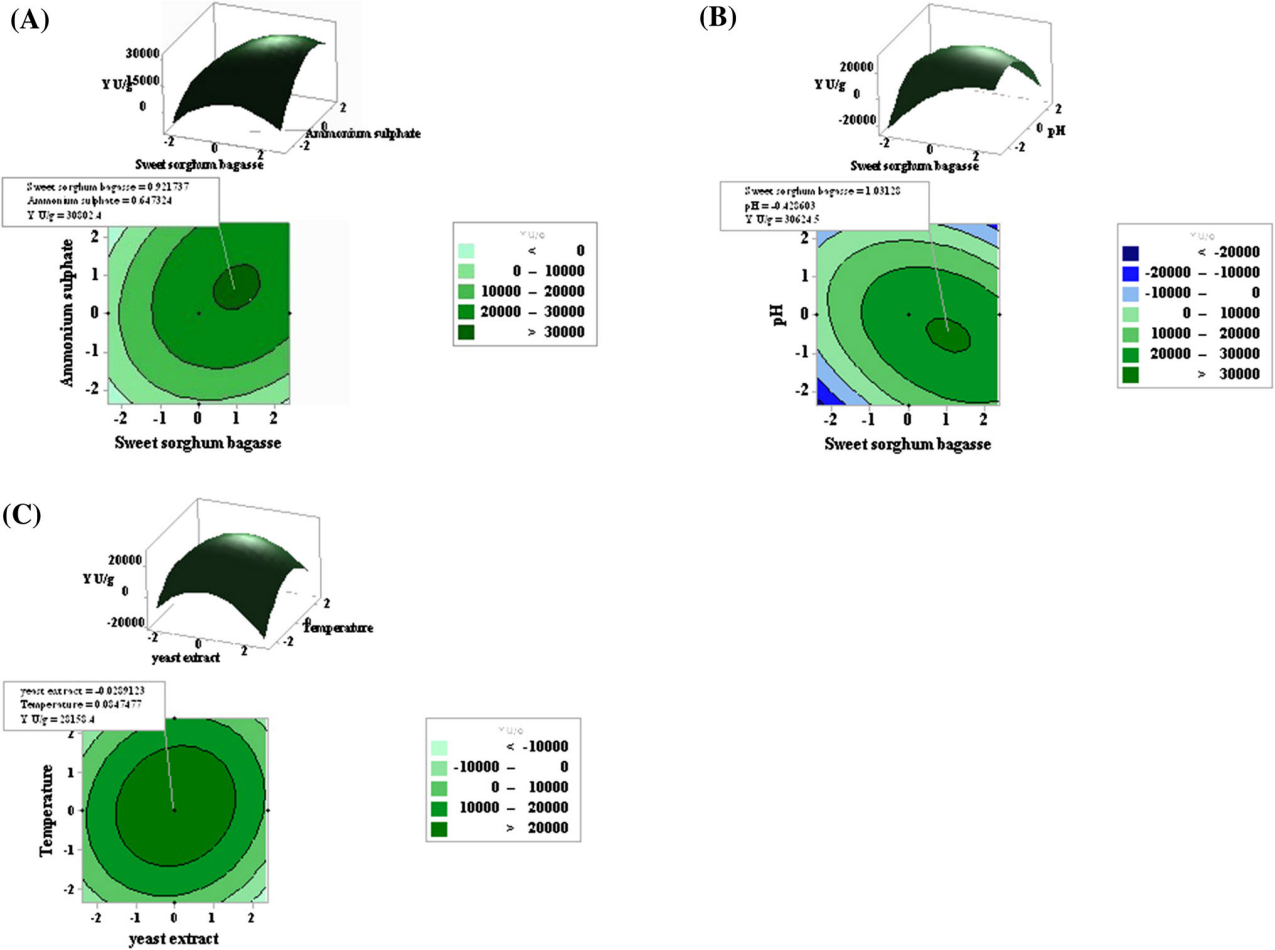
3D response surface and contour plots showing interactions between independent variables. Sweet sorghum bagasse vs ammonium sulphate (**a**), sweet sorghum bagasse vs pH (**b**) and temperature vs yeast extract (**c**) affecting the xylanase production (U/g)

3D response surface and contour plots were generated to investigate the interaction among the variables and to determine the optimum concentration of each variable for maximum xylanase production by *P. citrinum* isolate HZN13. Interactions between two factors were studied while keeping the other factor at middle level. Significant interactions (*p* < 0.05) were observed between sweet sorghum bagasse and ammonium sulphate, sweet sorghum bagasse and pH, and yeast extract and temperature. Value of *p* > 0.05 for *AB*, *AE*, *BC*, *BD*, *CD*, *CE* and *DE* (Table 5) indicated insignificant interactions. The negative value of coefficients for *D*, *A* ^2^, *B* ^2^, *C* ^2^, *D* ^2^, *E* ^2^, *AB*, *AD*, *CD* and *DE* indicates that xylanase production was affected at points other than optimal point. Maximum xylanase production occurred with sweet sorghum (20 g/50 ml) and ammonium sulphate (0.34–0.36 %) concentrations as shown in Fig. 3a. Higher concentration of sweet sorghum bagasse (25–30 g/50 ml) and pH 4–4.5 showed higher xylanase titers (Fig. 3b). With yeast extract concentration range of 0.4–0.6 % and higher temperatures between 35 and 40 °C xylanase titers were higher (Fig. 3c). High levels of xylanase production with increased sweet sorghum bagasse concentration could be due to the fact that sweet sorghum bagasse is an efficient nutrient for xylanolytic microorganisms as it constitutes of hemicellulose and substituted insoluble xylan. The higher production of xylanase may also be attributed to the better expression of multiple xylanases (Fig. 2b) produced by *P. citrinum* isolate HZN13 using sweet sorghum bagasse. Decrease in xylanase production at higher sweet sorghum bagasse levels (30 g/50 ml) may be due to the formation of a thick suspension and also due to the accumulation of reducing sugars. The maximum xylanase yield with different concentrations of the factors can be visualized from the contour plots where the surface of the smallest ellipse indicates the maximum yield. The elliptical contour plots indicate strong interaction between the variables (Fig. 3). Additional experiments were carried out in triplicate to validate the optimal predictions of the model. Xylanase production under optimized conditions (sweet sorghum bagasse 25 g/50 ml; ammonium sulphate 0.36 %; yeast extract 0.6 %; pH 4; temperature 40 °C) yielded 30,144 U/g as compared to predicted yield of 30,675 U/g. A 3.14-fold increase in xylanase production was obtained after statistical optimization. The verification revealed high model accuracy indicating the model validation under the tested conditions. However, we report a significantly high-level xylanase (30,144 U/g) optimized production from *P. citrinum* isolate HZN13. A higher fold increase in xylanase production would be further possible by considering other physical parameters that have an effect on xylanase production such as substrate-to-moisture mass ratio, incubation time, and inoculums concentration. Substrate-to-moisture ratio is an important process variable which is known to significantly affect many SSF processes. Ghoshal et al. (2012) reported 151.7 U/g (unoptimized conditions) and 1645.3 U/g (optimized conditions) of xylanase activity from *P. citrinum* by SSF. A positive effect of wheat bran substrate concentration and physical parameters during optimization has been studied previously in *Trichoderma reesei* SAF3 with enhanced xylanase yield of 299.7 U/g from 75 U/g with a 3.9-fold increase (Kar et al. 2013). A similar 3- and 3.1-fold increase in xylanase optimization is also evidenced by *Streptomyces* sp. P12–137 and *C. cellulans* CKMX1, respectively, using chemical parameters (Coman and Bahrim 2011; Walia et al. 2015). An optimized medium comprising of only chemical process parameters also showed threefold increase in xylanase by similar, nonlinear relationships between the independent variables affecting endoxylanase and endoglucanase production has been studied in *Penicillium janthinellum* (Kundu et al. 2012). RSM has been successfully used in the optimization of medium compositions for xylanase production (Biswas et al. 2010).

### Xylanase immobilization and its stability

**Fig. 4:**
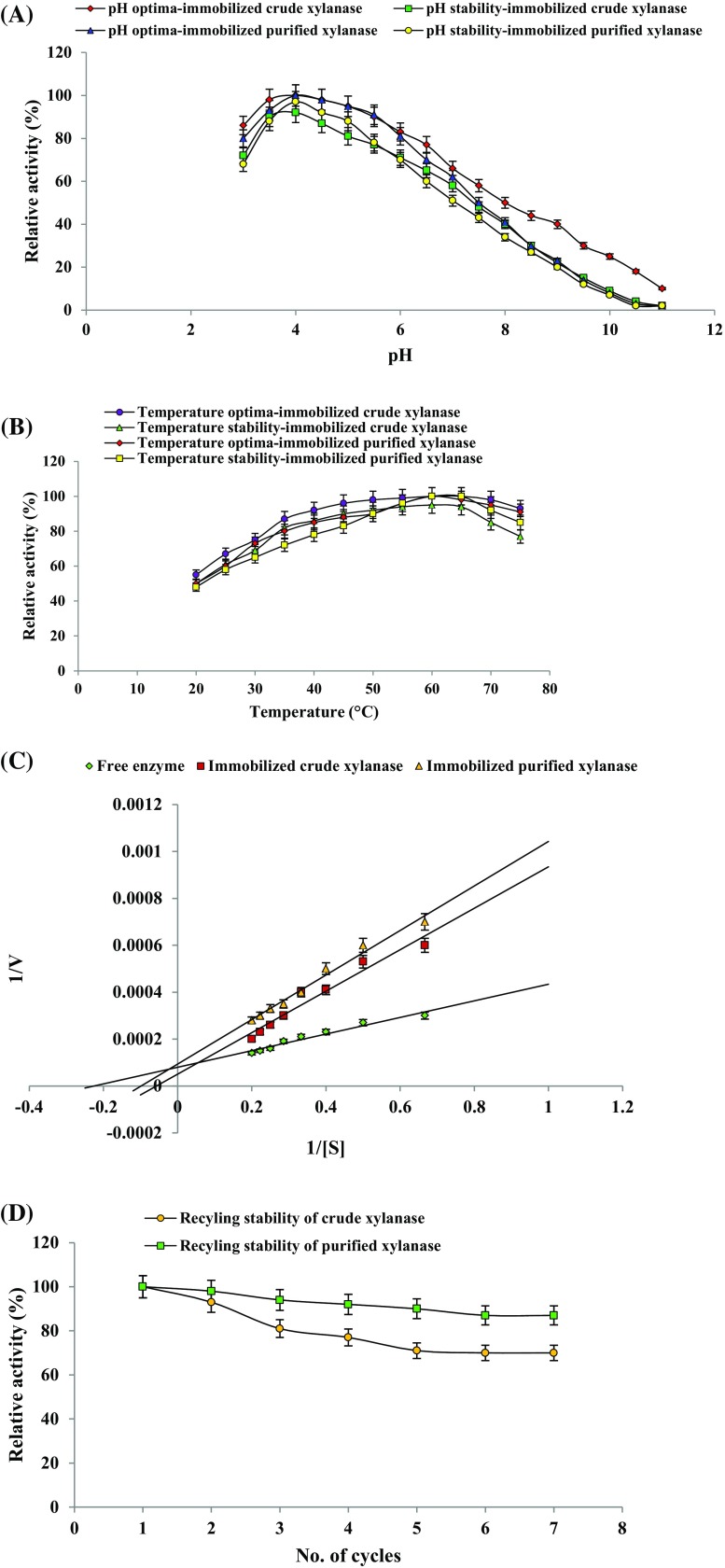
pH optima and its stability (**a**), temperature optima and its stability (**b**), enzyme kinetics (**c**) and recycling stability (**d**) of glutaraldehyde-activated calcium-alginate immobilized crude and purified xylanase

Currently, research on xylanase has gained much attraction due to their diverse applications in industry. Industrial processes largely utilize immobilized enzymes as they can be reused thereby reducing the production costs. In this study, the xylanase produced from *P. citrinum* isolate HZN13 was immobilized on glutaraldehyde-activated calcium-alginate beads as it is easy and economical. The glutaraldehyde-activated calcium-alginate immobilized crude and purified xylanase showed 9850 and 6100 U/ml of initially activity thereby retaining 65 and 88 % of activity after immobilization. Comparatively, the immobilization efficiency of immobilized purified xylanase was better and showed better stability. The reduction in the activity may be related to the fact that xylan being a large polymer might have poor diffusibility into the beads and low accessibility to enzyme active sites thereby decreasing the activity. But, the process is advantageous as the product diffusion is faster thereby reducing the risk of product inhibition. The glutaraldehyde-activated calcium-alginate immobilized crude and purified xylanase was reused for seven successive cycles retaining 70 and 87 % of its initial activity (Fig. 4d). Comparatively, a 23 and 8 % loss in initial activity was observed up to fourth cycle from immobilized crude and purified xylanase, respectively, suggesting the better stability of immobilized purified xylanase. The loss in activity could be due to enzyme inactivation during repeated recycling. Immobilization processes has been used to improve the stability of the enzymes. Earlier studies on reusability of xylanase by immobilization on glutaraldehyde-activated aluminum oxide pellets (Nagar et al. 2012) and glutaraldehyde–alginate beads (Pal and Khanum 2011) have been demonstrated with progressive loss in activity as the no. of cycles increases.

### Characterization of immobilized xylanase

Immobilization alters the enzyme characteristics and performance; therefore, a study on immobilized enzyme characterization is necessary. Glutaraldehyde-activated calcium-alginate immobilized crude and purified xylanase showed optimum activity in acidic pH range of 3.0–5.5 and 4.0–5.0, respectively (Fig. 4a) and temperature range of 45–75 °C and 60–75 °C, respectively (Fig. 4b). In our previous studies, free enzyme was also found to show maximum activity in acidic pH range of 3.5–5.0 and temperature range of 55–75 °C. However, higher activities at optimum pH and temperature range resulted from immobilized purified enzyme in comparison to free enzyme and immobilized crude enzyme, but showed a narrower pH and temperature optimum range. A higher pH stability of 97, 92 and 88 % (relative activity) was observed at pH 4.0, 4.5 and 5.0, respectively, with immobilized purified enzyme (Fig. 4a). In addition, higher temperature stability between 60 and 75 °C was revealed for immobilized purified xylanase (Fig. 4b) in comparison to immobilized crude xylanase. The increased pH and temperature stability of immobilized xylanase over free enzyme prove to be advantageous for industrial processes. Similar to our observations, displacement of pH and temperature for immobilized enzymes has been observed in earlier studies (Nagar et al. 2012; Pal and Khanum 2011) and variations in the displacements may be due to changes in matrix type and with the type of interaction between the enzyme and matrix. A similar increase in the optimum temperature of immobilized partially purified enzyme was reported (Sen et al. 2012). Kinetics of enzyme revealed a *K*_m_ of 4.76, 12.5 and 11.11 mg/ml and *V*_max_ of 7100, 12,500 and 10,000 U/mg by free, immobilized crude and immobilized purified enzyme, respectively (Fig. 4c). *K*_m_ value of immobilized enzymes were higher as compared to free enzyme indicating lower substrate affinity possibly due to steric hindrance of the active site for substrate binding by the matrix. A profound increase in *V*_max_ value of immobilized enzymes could be attributed to the hindrance in the conformational changes of enzyme and also due to variations in the properties of active site for interactions between enzyme and matrix (Asgher et al. 2012; Wang et al. 2014). However, immobilized purified enzyme showed a better catalytic efficiency in comparison to immobilized crude enzyme. A similar increase in *K*_m_ and *V*_max_ values has been observed in previous studies (Nagar et al. 2012; Pal and Khanum 2011). Decreased enzyme affinity and lower *V*_max_ was reported in case of immobilized partially purified enzyme possibly due to shielding effect of the entrapment (Sen et al. 2012).

### Enzymatic hydrolysis and HPLC analysis of hydrolysed substrate

**Fig. 5:**
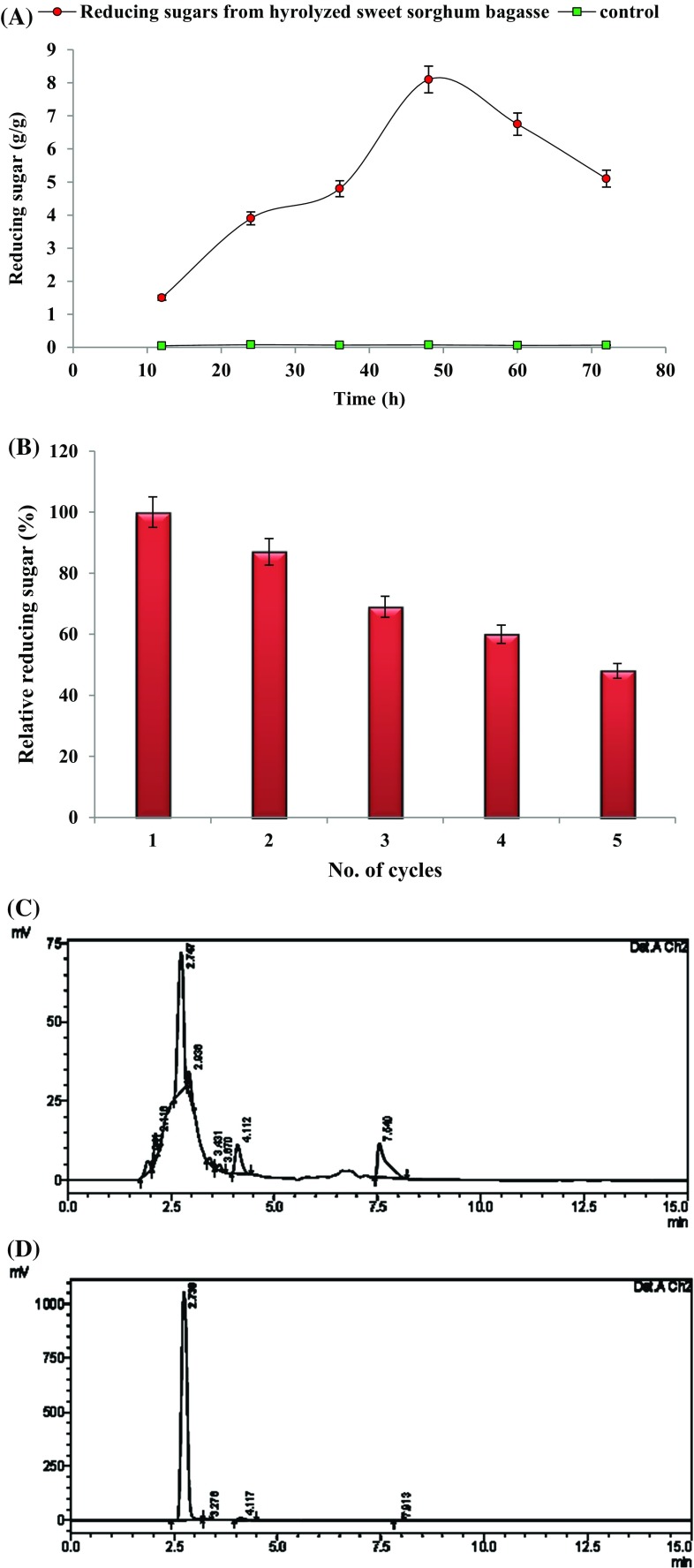
Enzymatic hydrolysis of sweet sorghum bagasse with glutaraldehyde-activated calcium-alginate immobilized xylanase (**a**), reusability of glutaraldehyde-activated calcium-alginate immobilized xylanase for enzymatic hydrolysis (**b**) HPLC chromatogram showing the profile of hydrolyzed products from sweet sorghum bagasse (**c**) and HPLC chromatogram for standard xylose (**d**)

Enzymatic hydrolysis of alkali-pretreated sweet sorghum bagasse with glutaraldehyde-activated calcium-alginate immobilized crude xylanase was carried out for the production of fermentable sugars. 8.1 g/g (g of reducing sugar per g of bagasse) of maximum reducing sugars was produced at 48 h (Fig. 5a). 6.4 g/g (48 h) of reducing sugars was found to be released with free enzyme in our previous studies. Immobilized crude enzyme was reused up to five consecutive cycles for enzymatic hydrolysis and >65 % of reducing sugars were produced up to three cycles (Fig. 5b). Enzymatic hydrolysis of xylan to xylooligosaccharides using immobilized xylanase on glyoxyl-agarose supports has been previously reported (Aragon et al. 2013a). The filtrate collected after the enzymatic hydrolysis was analyzed for the presence of xylose sugars by HPLC. Figure 5c illustrates the HPLC analysis of hydrolyzed products from sweet sorghum bagasse. Peak with RT of 2.7 (Fig. 5c) was confirmed as xylose in comparison to the standard (Fig. 5d). Other peaks with RT of 2.1, 2.9, 3.6, 4.11 and 7.5 (Fig. 5c) may be predicted to be any of the xylooligosaccharides (xylobiose, xylotriose, xylotetraose, xylopentaose or xylohexaose) which are commonly produced during hydrolysis of xylan-containing substrates. Similar study has been reported with corn cobs for xylanase produced by *T. koeningi* (Bandikari et al. 2014).

### SEM and EDX analysis

SEM analysis illustrates that the morphological changes in the bagasse occurred during hydrolysis by *P. citrinum* isolate HZN13. As compared to the SEM image of untreated sweet sorghum bagasse (control) from previous report (Bagewadi et al. 2016), which showed a closed unique structure of the fibers suggesting the recalcitrant nature, the SEM image of sweet sorghum bagasse hydrolyzed by *P. citrinum* isolate HZN13 showed fungal mycelia structures adhered to the substrate particles as seen in Fig. S1A (Supplementary information, SI). Enhanced porosity of the bagasse due to hydrolysis is evidenced. This indicates the consumption of hemicellulosic material by fungi during saccharification. Removal of the hemicellulosic fraction by xylanase produced by *P. citrinum* isolate HZN13 further improves the digestibility of biomass. Sugar production during saccharification of biomass by fungi can be employed for bio-ethanol production. In accordance with previous reports, SEM micrographs have been used to evaluate the effect of dilute acid pretreatment on the structural characteristics of the sugarcane lignocellulosic biomass (Pereira et al. 2016) and also to study the pretreatment effects on morphology of pine wood (Wi et al. 2015).

The elemental studies of treated (hydrolyzed by *P. citrinum* isolate HZN13) sweet sorghum substrate was performed by SEM/EDX. The biomass has various elements that vary during the hydrolysis process. The SEM/EDX analysis of *P. citrinum* isolate HZN13-hydrolyzed sweet sorghum bagasse is shown in Fig. S2 (SI). The carbon content of untreated bagasse (67.85 %) from previous study (Bagewadi et al. 2016) was progressively reduced to 61.58 % during hydrolysis. Hence, it is evident that the fungus consumes the carbon for xylanase secretion. The utilization of nitrogen for the growth and enzyme production by the organism is evidenced by reduction in nitrogen content during the course of hydrolysis. The occurrence of Mg^2+^ and Ca^2+^ in hydrolyzed bagasse sample may be due to their accumulation by fungal cell on the surface during growth and xylanase secretion. The conformational stability of xylanase is maintained by these ions by binding to the non-catalytic xylan binding site, which is involved in substrate hydrolysis. EDX has been previously used to study the fungal mycelia elemental composition during the growth on wheat bran (Kar et al. 2013).

### FTIR analysis

FTIR tool was employed to investigate the structural and functional group changes of *P. citrinum* isolate HZN13-hydrolyzed sweet sorghum bagasse and compared with the control (untreated sweet sorghum bagasse) from previous report (Bagewadi et al. 2016). The spectrum of *P. citrinum* isolate HZN13-hydrolyzed sweet sorghum bagasse is shown in Fig. S3 (SI). Hydroxyl group stretching enhanced after hydrolysis of bagasse. The deprivation of fibrillar structure of hemicellulose is seen after hydrolysis. An amine N–H stretch peak is found around 3419 cm^−1^ in control and hydrolyzed sample. A C–H stretching is depicted around 2915 cm^−1^ due to destruction of aliphatic groups during hydrolysis process. A stretch of urethane amides was noticed around 1734 cm^−1^ range in control and hydrolyzed bagasse. An alkene C–C symmetric stretch was detected around 1657 cm^−1^ range in hydrolyzed bagasse. A stretch of lignin aromatic ring with aromatic C=C bending was examined in range of 1600–1500 cm^−1^ in control. Disappearance of these peaks in hydrolyzed bagasse indicates the delignification progression. A weak C–O stretching around 1450–1400 cm^−1^ was observed in both the samples. Peaks in the region of 1315 cm^−1^ are associated to C–H and C–O stretching of acetyl group in hemicelluloses in varying intensities in both the samples. In the vicinity of 1161 cm^−1^, a C–O or C–O–C stretching was witnessed suggesting the occurrence of cellulose and hemicellulose structures. The peaks near 1100–1050 cm^−1^ were credited to β-(1–3) polysaccharides in hydrolyzed bagasse. An increase in the intensity of the peaks around 856 and 800 cm^−1^ was recognized for cellulose following hydrolysis (Adapa et al. 2011; Bagewadi et al. 2016). Thus, the FTIR spectra demonstrated the degradation phenomenon of bagasse by *P. citrinum* isolate HZN13. FTIR spectroscopy is commonly employed to examine the structure and the chemical changes in lignocellulosic biomass during pretreatment and hydrolysis. Earlier report on FTIR characterization of lignocellulosic biomass also suggests similar chemical changes (Adapa et al. 2011). Wi et al. (2015) also used FTIR to study the breakdown of the aromatic structure of lignin caused during pretreatment. Similar changes were induced in sugarcane lignocellulosic biomass varieties after the dilute acid pretreatment and were confirmed by FTIR (Pereira et al. 2016). These studies demonstrate efficient biomass consumption for high titers of xylanase production from *P. citrinum* isolate HZN13.

## Conclusion

In the present study, the *P. citrinum* isolate HZN13 isolated from forest soil produced exceptionally high-level cellulase-free xylanase from a variety of agro-waste residues. Xylanase production using sweet sorghum bagasse was statistically optimized by PBD and RSM-CCD. Glutaraldehyde-activated calcium-alginate immobilized xylanase showed pH and temperature stability with increased kinetics as compared to free enzyme and was used efficiently for enzymatic hydrolysis of bagasse. Very few reports on high yields of xylanase by *P. citrinum* isolates are available. SEM, EDX and FTIR characterization of bagasse presents the insights into hydrolysis process. Xylose detection by HPLC from bagasse reveals the industrial significance of xylanase.

## Electronic supplementary material

Below is the link to the electronic supplementary material.

Supplementary material 1 (DOCX 222 kb)
