# Supplementary material for: Xylanase production from Penicillium citrinum isolate HZN13 using response surface methodology and characterization of immobilized xylanase on glutaraldehyde-activated calcium-alginate beads
Source: 3 Biotech. 2016 Aug 11;6(2):164. doi: 10.1007/s13205-016-0484-9 (PMC4980835; doi:10.1007/s13205-016-0484-9)
Supplement: Supplementary file 1 — Supplementary material 1 (DOCX 222 kb) [file 13205_2016_484_MOESM1_ESM.docx]

**Supplementary Information**

**Xylanase production from *Penicillium citrinum* isolate HZN13 using response surface methodology and characterization of immobilized xylanase on glutaraldehyde activated calcium-alginate beads**

Zabin K. Bagewadi^1^, Sikandar I. Mulla^1^, Yogesh Shouche^2^ and Harichandra Z. Ninnekar ^1,^*

^1^Department of Biochemistry, Karnatak University, Dharwad, Karnataka 580 003, India

^2^ National Centre for Cell Science, Pune University Campus, Ganeshkhind, Pune, Maharashtra 411 007, India

^*^Corresponding author’s address: Dr. H Z.Ninnekar,

Professor,

Department of Biochemistry,

Karnatak University,

Dharwad 580 003, Karnataka, India.

Tel.: +91-0836-2215243; fax: +91-0836-2747884.

Email:[hzninnekar@yahoo.com](mailto:hzninnekar@yahoo.com)

**Supporting Information: 4 Pages, 3 Figures**


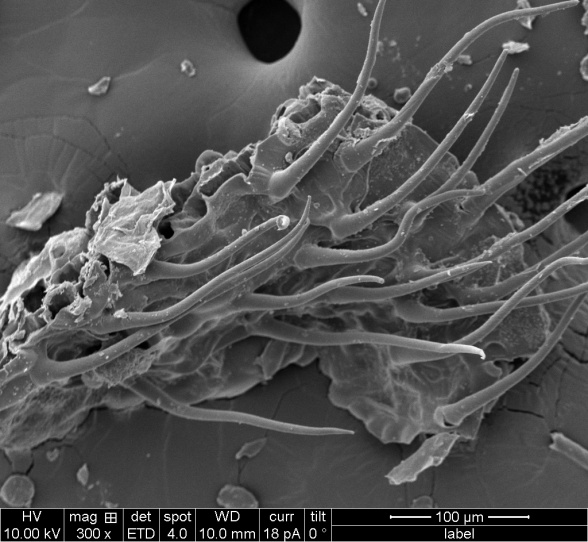


**Fig. S1.** SEM analysis of alkali pretreated sweet sorghum bagasse hydrolyzed by *Penicillium*

*citrinum* isolate HZN13


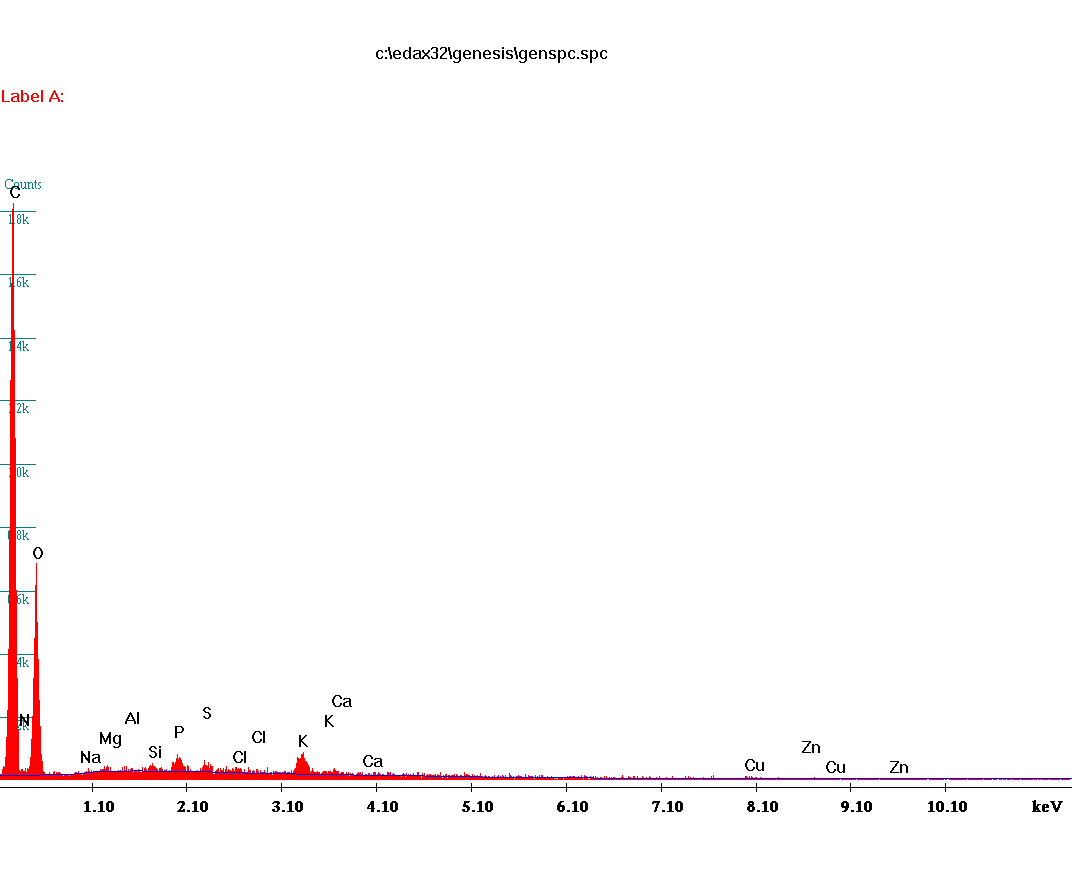


**Fig. S2.** EDX analysis of alkali pretreated sweet sorghum bagasse hydrolyzed by *Penicillium citrinum* isolate HZN13


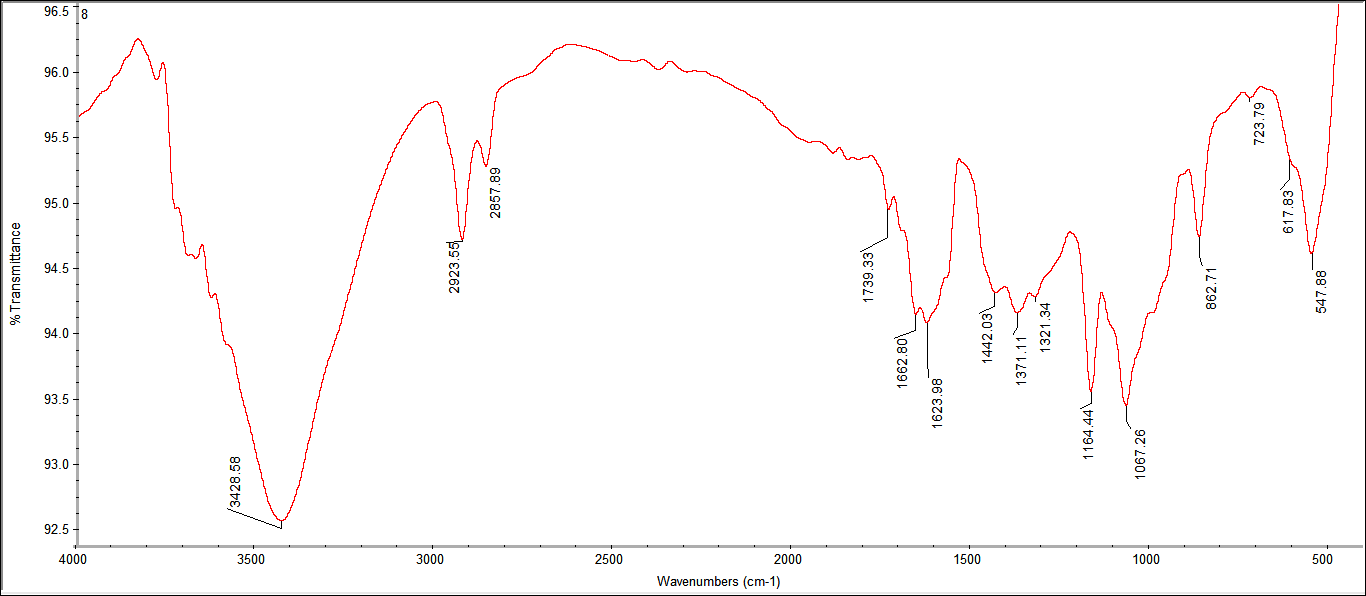


**Fig. S3.** FT-IR analysis of alkali pretreated sweet sorghum bagasse hydrolyzed by *Penicillium citrinum* isolate HZN13
